# Issues and Perspectives for the Study of Disruptive Clinician Behavior

**DOI:** 10.7759/cureus.63314

**Published:** 2024-06-27

**Authors:** Manabu Fujimoto

**Affiliations:** 1 Institute for Teaching and Learning, Ritsumeikan University, Kyoto, JPN

**Keywords:** influence mechanism, occurrence mechanism, hierarchical structure, definition and attribution, disruptive clinician behavior

## Abstract

This article discusses issues and perspectives related to the study of disruptive clinician behavior (DCB) to improve patient safety and healthcare professionals’ work environments. Multiple terminologies and ambiguous definitions have resulted in conceptual confusion in studies on DCB. In addition, subjective classifications have led the attributes of DCB to overlap and fluctuate. Therefore, we share Rosenberg’s definition of DCB as “any inappropriate behavior, confrontation, or conflict, ranging from verbal abuse to physical and sexual harassment.” It is recommended that DCB be understood as a hierarchical structure identified through statistical analysis of field survey data. Furthermore, a recurring list of items is duplicated across existing studies on DCB triggers, contributing factors, and influences. These items can be organized into separate path models based on their mutual relationships. Given these assumed models, we believe that further studies on DCB can shift toward elucidating the mechanisms of occurrence and impact. Finally, based on the path models, we recommend improving healthcare professionals’ psychological and social states through a policy shift from “zero-tolerance” to “to err is human” as a priority issue for DCB prevention and countermeasures.

## Introduction and background

Medical staff members should perform their duties with a high level of professionalism to provide safe and high-quality medical care [1]. In addition, communication plays a central role in team medicine [2], and developing collaborative and supportive relationships is essential. For example, the high-risk aviation industry maintains a high level of safety based on a reliable organizational structure [3]. In the medical industry, highly specialized members of the medical staff should cooperate on an equal footing and with respect for each other to perform tasks and make decisions, with solving the problems of patients and their families as the top priority. However, medical staff members often exhibit behaviors and attitudes that interfere with the provision of team care and make others uncomfortable. This behavior is referred to as disruptive clinician behavior (DCB) [4], and many medical staff members have been victims of it [5,6]. Rosenstein, who has attempted to clarify the definition of DCB since the early 2000s, describes it as “any inappropriate behavior, confrontation, or conflict, ranging from verbal to physical abuse and sexual harassment” [4,7].

Following the publication of the Institute of Medicine’s report titled “To err is human” [8] in 1999, the importance of patient safety has been recognized worldwide. Interest in DCB has grown among US healthcare organizations because of its impact on the recruitment and retention of medical staff and the quality and safety of patient care. In 2008, the American Medical Association (AMA) defined DCB as “a measure of personal conduct, verbal or physical, that has the potential to negatively affect patient care or the ability to work with other members of a healthcare team” [9]. Furthermore, the AMA included a procedure for disciplining DCB in the medical staff code of conduct under a zero-tolerance policy [10]. This principle has led to active countermeasures to eradicate DCB in the United States. However, many medical staff members and institutions were unable to adequately deal with DCB owing to a lack of information and passivity [11]. Therefore, DCB continues to affect patient safety, the work environment, and healthcare outcomes [12], making it a pernicious and persistent problem in the healthcare field [13]. This article discusses the challenges and research prospects in discussing DCB by reviewing previous findings on the concept, attributes, and mechanisms of DCB incidence and its impact. The article aims to contribute to the development of research aimed at the prevention of and countermeasures for DCB.

## Review

Concepts and attributes of DCB

Many similar concepts related to DCB have hindered academic research and impacted the accumulation of knowledge toward eradicating DCB [14]. Medical literature addressing disruptive physicians appeared in 1978 [15]; however, the concept of DCB was first mentioned in the literature in 1995 [16]. Since then, various researchers and institutions have proposed different definitions of DCB [17]. The problem of a proliferation of definitions was highlighted in 1999 [18]; defining a concept unambiguously to enable communication among researchers is the first step in scientific research. Therefore, the plurality of the DCB concept should be viewed in terms of hierarchy and discriminability.

DCB is a superordinate concept that encompasses various behaviors and can be subdivided into multiple categories and subcategories depending on the specific type of behavior. For example, Walrath et al. used semi-structured group interviews to identify three categories of DCB, namely, incivility, psychological aggression, and violence [19]. In a literature review, Oliveira et al. [17] also identified incivility, psychological violence, and physical or sexual violence. Petrovic and Scholl [14] classified the 207 words they had extracted from 163 articles into eight categories. The category that contained the most diverse terminology was passive-aggressive behavior (129 terms), which included intentional neglect and uncooperative behavior [20]. This was followed by physical or verbal threats and abuse (25 each), physical violence and harassment (18 each), intimidation (nine), and bullying and discrimination (eight each). Although several studies worldwide have attempted to classify DCB [17,21,22], the categories are not necessarily consistent. This conceptual confusion has been exacerbated by studies that treat sub-concepts or specific behaviors as synonymous with DCB, including incivility [23], bullying [24], and violence [25]. In addition, several articles have incorporated the findings of studies targeting specific behaviors included in DCB into discussions of the higher-level concept of DCB without noting these specific behaviors.

Furthermore, DCB is sometimes described using other terms. Doctors at the top of the professional hierarchy often demonstrate DCB to other medical staff [7,12,20,26]. Many studies have focused on disruptive physician behavior [20,27] and disruptive surgeon behavior [28], thereby only recognizing doctors as the perpetrators, even though nurses, paramedics, and medical office staff could also act as one. Limiting this behavioral pattern to doctors is inappropriate unless for a specific reason. Additionally, other studies have also used the term “unprofessional behavior,” which has traditionally been used in medical student education, as DCB is among the attitudes and behaviors that violate the professional ethics medical staff members are expected to follow [13,29]. However, our concern is with the quality and safety of medical care. Therefore, we focused on inappropriate interpersonal behavior, which diminishes affinity and trust between victims and others and damages relationships, thereby resulting in poor communication and teamwork and compromising the quality of care and patient safety. Behaviors that disrupt relationships among medical staff, constituting DCB, violate the code of medical ethics [30] and should be observed by professionals. Therefore, unprofessional behavior is a superordinate concept encompassing DCB and is not synonymous with it. Additionally, the term DCB should be used more narrowly in studies focusing on the aggravation of the victim’s psychological and social adaptation.

Hierarchical Categories With a Quantitative Approach

Various issues with the multiple terms for DCB can be attributed to differences in researchers’ interpretations. The meta-approach, which reviews existing studies, and the qualitative approach, which conducts content analysis, strongly reflect the impact of analyst subjectivity. Conversely, quantitative approaches quantify the respondents’ perceptions and classify them using statistical analysis. Although there has been only a small amount of previous research using this approach, Rehder et al. [31] selected six items that were representative of DCB and conducted a questionnaire survey. Through a confirmatory factor analysis, a one-factor structural model was created, in which three pairs were categorized as “bullying others-humiliation of others,” “turned back-hung up phone,” and “discriminatory comments-physical aggression.” Fujimoto et al. also conducted an open-ended questionnaire along with two questionnaires on medical staff members’ perceptions of DCB. Their analysis, based on a scale construction process, identified the hierarchical categories of DCB [32]. This hierarchical model (Table 1) comprised categories also identified in many subjective studies. DCB can be broadly divided into “interpersonal aggression” and “job-related aggression.” However, some studies on job-related aggression [33,34] included “passive aggression” (intentional negligence by subordinates) and “mismanagement practice” by superiors. Mismanagement practice was further divided into “non-supportive coercion” (e.g., forced complicity in business or fraud) and “arbitrary decision-making” (e.g., unilateral policy-making without consultation). The Joint Commission also mentioned passive aggression in its Sentinel Event Alert 40 [35], which indicates that a comprehensive discussion of DCB, including job-related aggression, is necessary.

**Table 1:** Attributes and hierarchical structure of disruptive clinician behavior (DCB). Numbers represent pairs of correlated DCB [32] in decreasing order of manifestation: 1 = silent aggression, 2 = personal aggression, and 3 = power harassment. The table is original.

Category	Factor	Sub factor	Pair
Interpersonal aggression	Psychological aggression	Intimidation	1
		Reproof	
		Threats	3
		Abusive language	2
	Incivility		2
	Ignoring		1
	Physical violence		
Job-related aggression	Mismanagement practice	Arbitrary decision	
		Nonsupportive coercion	3
	Passive aggression		

Interpersonal aggression involves “psychological aggression,” “incivility,” “ignoring,” and “physical violence.” Psychological aggression is further divided into “intimidation,” “reproof,” “threats,” and “abusive language.” The 10 identified DCB types roughly corresponded one-to-one or across several types with the category system established by Petrovic et al. [14]. However, the category systems established by Petrovic et al. [14] and Fujimoto et al. [32] have two differences. The first is discrimination. In the classification used by Fujimoto et al. [32], discrimination is an item that constitutes abusive language, whereas Petrovic et al. [14] consider it an independent category. This may be because discrimination against racial, ethnic, religious, and other minorities occurs more frequently in the West, where diversity is higher than in Japan [36]. The second is harassment, which Petrovic et al. [14] classified as an inclusive harassment category based on the commonality of languages. However, power and sexual harassment, for example, are essentially different behaviors in terms of their cause, conduct, and impact. The classification by Fujimoto et al. [32] leaves no room for the classifier’s subjectivity or commonality of language in the classification criteria. Therefore, in their classification, power harassment refers to a pair of behaviors: mismanagement practices and reproofing. Thus, power harassment is a DCB in which a superior imposes unreasonable words and actions on a subordinate based on their authority. Sexual harassment is limited to the number of victims and has only been identified as an independent category in studies on DCB victimization among nursing students [37].

Axis of Observable Behaviors

A different classification criterion for interpersonal aggression is “observable” [38,39]. Dichotomizing specific behaviors, physical violence, threats, abusive language, and ignoring are transient, explicit types of DCB that cause outbursts of frustration. Simultaneously, intimidation, reproofing, and incivility are spiteful types of DCB repeatedly and discreetly directed at specific individuals over a long time on an ongoing basis. The three DCB pairs (Table 1) corresponding to the pairs in the model by Rehder et al. [31] are silent aggression, personal aggression, and power harassment. Hastie et al. noted that DCBs range from incivility (e.g., yelling without malice, not cooperating, superficial listening, and nitpicking) to microaggressions (e.g., making jokes about identity, slander, and stereotypical misperceptions of competence). Although each of these occurs separately and has individual adverse influences, microaggressions, such as making jokes about identity, slurs, and stereotypical misperceptions of competence, may escalate to bullying and harassment with clear malicious intent [40]. When covert trivial attacks occur repeatedly, they gradually become malicious and blatant attacks with a clear harmful intent.

Plurality of Definitions

Conceptual confusion hinders progress in academic research. Therefore, researchers must understand the hierarchical structure of DCB, be aware of whether their interests are inclusive or specific, and use these terms discriminately and correctly. This also applies to the several definitions of DCB. Most existing definitions include the long modifier “inappropriate behavior.” However, the content of “inappropriate behavior” varies widely, as described above. In addition, the typical content of the long modifier is to exacerbate the victim’s psychological and social adaptation, thereby impeding their motivation and commitment and reducing their performance. Inappropriate behavior can lead to disagreements and affect workplace relationships, including relationships between the perpetrator and victim and those around them, inhibiting communication and teamwork. These impacts go beyond the intrapersonal and interpersonal levels as they also put patient safety and overall organizational healthcare quality and management at risk. As described above, many existing definitions define DCB as attributes consisting of various specific behaviors and causal influences that affect different aspects of healthcare services through the influence processes comprising the multiple steps described above. Rosenstein’s definition of “any inappropriate behavior, confrontation, or conflict, ranging from verbal abuse to physical and sexual harassment” [4] is regarded as the clearest and most comprehensive [17]. The plurality of the DCB concept stems from the different definitions proposed by researchers; therefore, future studies should only use Rosenstein’s definition.

Occurrence mechanism of DCB

The Johns Hopkins model, which describes the components associated with DCB, identifies three categories of DCB triggers, namely, intrapersonal, interpersonal, and organizational factors [41]. Previous studies have described the causes of DCB as triggers or contributing factors. This study makes a conceptual distinction between triggers and contributing factors: “triggers” are defined as “reasons” that trigger DCB, whereas “contributing factors” are considered as factors that influence the process by which the perpetrator expresses DCB because of triggers. The mechanism encompassing these factors is the occurrence mechanism of DCB.

Intrapersonal Factors

Personality, which includes sociability, trustworthiness, and empathy, is a stable trait associated with DCB. It has been reported that medical staff members who are more likely to be victimized have introverted, conscientious, neurotic, and submissive personalities [42]. Therefore, a low prosocial personality poses problems for both perpetrators and victims. Other identified contributing factors include self-control, impulse control, stress control, communication and social skills, and pessimistic sensitivity [26,43,44]. Personality traits such as type A personality, narcissism, or passive aggression tendencies also increase the risk for DCB [45].

The temporary status of medical staff members also plays an important role in the occurrence of DCB. Overwork and irregular shift rotations are major burdens on medical staff [46]. Stress induces DCB by temporarily decreasing empathy, impulse control, and conflict-coping skills [45,47], and many doctors who accumulate stress to the point of burnout may express DCB. In addition, inexperienced medical staff members may not adapt well to patients and situations and are more likely to feel rushed and stressed in their work. The perception of immaturity may also mean that inexperienced staff members avoid interacting with other medical staff members and respond inappropriately [17]. Psychiatric disorders (e.g., depression, addiction, stress, and burnout) have also been implicated. However, it has been argued that mental illness should not be considered a contributing factor to DCB, as the individual is not responsible for it [20].

Medical staff members’ demographics also contribute to the incidence of DCB. For example, the tendency toward DCB differed between male-dominated doctors and female-dominated nurses [33,48]. Other attributes include ethnicity and work experience, whereby majorities tend to express DCB toward minorities [23].

Interpersonal Factors

DCB affects the relationships, communication, and teamwork among medical staff members, and deterioration of these factors is a regression trigger for DCB [17]. In addition, medical staff members may become frustrated and stressed when their needs and expectations of others are not met or they are betrayed [49]. Similarly, strong beliefs about health care may create ill feelings, whereby a person will not tolerate others who contradict them. However, it should be noted that DCB is counterproductive as it alters others’ behavior in a certain desired direction. The aforementioned individual demographics and organizational hierarchy (discussed below) can also be considered interpersonal factors when viewed as differences between perpetrators and victims.

Organizational Factors

In one study, four of the five causes of DCB identified through interviews with medical staff members were organizational factors, namely, organizational mindset, management, work conditions, and education and training [50]. Among these, organizational culture and mindset are particularly significant. Deviations from rules and norms are often normalized in healthcare organizations [51]. Similarly, in organizations where DCB is overlooked, perpetrators feel less guilty, and DCB may become the norm. Organizations can be particularly permissive toward doctors, who generate significant revenue for the healthcare organization [35,52], which fosters an organizational culture that tolerates doctors’ DCB.

The hierarchy of authority in the hospital system is a major focus of DCB [6,7,12,26,53]. DCB can be expressed from supervisors to subordinates, supervisors to instructional students, doctors to other types of positions, and skilled to unskilled workers. In addition, horizontal DCB is more likely to occur between people in socially oppressed groups, such as those in the nursing profession [54]. Passive aggression can also occur from subordinates toward superiors. Other hierarchies include those based on race [55] and gender [56]. Non-sympathetic and non-accepting attitudes stemming from differences in experiences and values may also create conflict among medical staff. Working conditions are another critical issue; medical staff members are frequently stressed in organizations with poor working conditions, such as poor workload management, physical environment, infrastructure, and resources [57], or inefficient or inappropriate organizational culture, leadership, and teamwork [58]. The ability to control DCB and make calm decisions is restricted when medical staff members are under pressure. Therefore, the risk of improvised DCB increases.

The healthcare industry has unique inherent systemic problems, such as the demand for increased productivity, cost containment requirements, and fear of litigation [59]. External environmental pressures, such as increased healthcare policy-related scrutiny, policy responses to healthcare problems, and responsibility for healthcare safety, also heavily burden medical staff [60,61]. In addition to poor working conditions, medical staff members may feel strong psychological strain during busy times, emergencies, and when performing critical procedures that affect patients’ lives [62,63]. High stress and pressure reduce self-control among medical staff, which may lead to a decrease in willpower [64]. This reduced margin in a person’s mental state increases the risk of improvised DCB as it limits the margin of control and calm judgment of the DCB.

**Figure 1:**
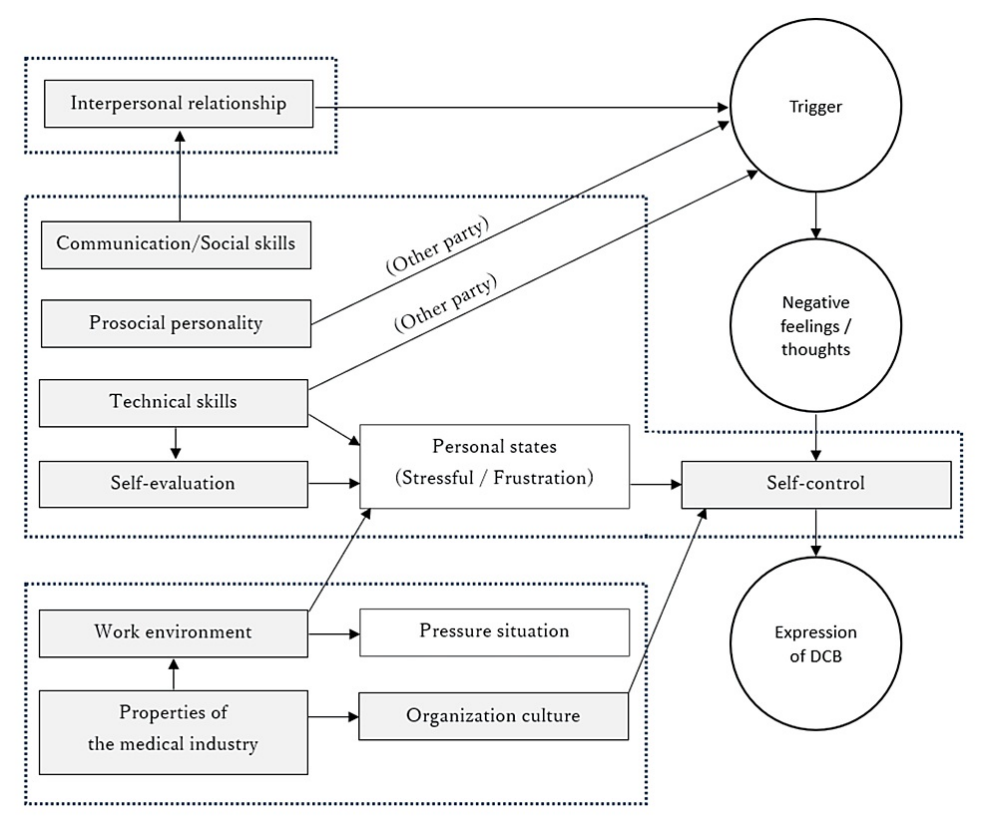
Model summarizing the findings on the occurrence mechanism of disruptive clinical behavior (DCB). The right side of the figure shows the flow of negative feelings and thoughts triggered by DCB and the expression of DCB to dissipate them, where self-control acts as a deterrent to this flow. The dotted boxes represent the interpersonal, intrapersonal, and organizational components from top to bottom. The light grey boxes represent the positive components that prevent the occurrence of DCB. The annotation (other party) indicates that these are triggers based on victim traits. The figure is original.

The bulwark model as an occurrence mechanism: When the findings on triggers and contributing factors cited in previous studies are organized as an occurrence mechanism for DCB, a causal relationship emerges. Organizational, environmental, and interpersonal problems aggravate the psychological state of medical staff, thereby facilitating DCB. Therefore, among intrapersonal factors, we should distinguish between perpetrators’ maladaptive traits and the worsening of their psychological state which is attributable to other causes. In addition, internal (e.g., hospital hierarchy) and external problems (e.g., unique characteristics of the medical industry) influence medical staff members independently. Based on these considerations, the DCB occurrence mechanism can be summarized in a multilayered model (Figure 1). The number of components in this figure is consistent with the number of references for the three factors (90, 33, and 68 references for individual, interpersonal, and organizational factors, respectively) [17]. Furthermore, a trigger is a situation in which a victim does not meet the perpetrator’s professional or interpersonal needs. Perpetrators also make arguments; by attending to these, we can uncover the details of triggers that remain unexplored. Conceptually, considering what are referred to as “triggers” in previous studies as contributing factors is appropriate. Several studies have examined these factors. In the hypothetical model of the occurrence mechanism (Figure 1), self-control can be likened to a bulwark that directs the current of negative feelings or thoughts caused by triggers downstream. If the negative feelings or thoughts caused by the trigger surpass an individual’s self-control, the bulwark may break and DCB will occur. Most contributing factors have adverse effects that make the bulwark vulnerable. Based on the concentration of paths in the bulwark model (Figure 1), we prioritized measures that would allow medical personnel to work with a relaxed mindset; thus, medical staff members working comfortably was our priority.

Influencing mechanism of DCB

The negative impacts of DCB have been extensively reported, including decreased safety and quality of care, increased risk of medical errors, deterioration of organizational culture, and increased difficulties in recruitment and retention [7,65,66]. The Johns Hopkins model lists three impact categories, namely, medical staff, patients, and organizational impact [41].

Impact on Medical Staff

A review by Oliveira et al. [17] showed that the literature most frequently mentioned impacts “for the worker or health team.” The top three impacts were mental distress, ranging from anxiety to burnout; interpersonal behavioral barriers, such as communication and teamwork; and job loss, in the form of layoffs and turnover. Other impacts included hostility toward the workplace, loss of work motivation, inability to concentrate, and isolation within the workplace, which may contribute to the top three impacts in terms of content. DCB adversely affects job satisfaction, patient satisfaction, quality of care, staff turnover, nurse shortages, and patient safety [33].

Impact on Patients

The most common impact on patients is decreased quality and safety of medical care, with the major outcome being patient dissatisfaction. DCB, which frequently occurs in high-pressure emergency departments [17], also has significant impacts on team dynamics, communication efficiency, information flow, and task accountability, all of which affect the medical staff. These issues affect patients in the form of adverse events, medical errors, patient safety violations, poor quality of care, and patient death [33]. A previous study showed that medical staff members in the emergency department were familiar with the stress, frustration, and loss of focus caused by DCB [67]. In addition, they understood its impact on patient safety: DCB resulted in poor quality of care and compromised patient safety, leading to medical errors, adverse events, and patient deaths. Surprisingly, they believed that adverse events caused by DCB could be prevented [33]. Many medical staff members in settings outside the emergency department also believe that DCB leads to medical errors and sometimes patient death [68]. In addition, almost all hospital administrators universally recognized that DCBs negatively affected medical care, to the patient’s detriment [69].

Impact on the Organization

Although there is a relatively small amount of literature addressing the impact of DCB on health organizations, many organizations are concerned about the financial burden arising from DCB, such as legal procedures, hiring new staff, and additional adverse event treatment; the deterioration of internal organizational dynamics and culture; and the degradation of their public image. Therefore, organizations are strongly affected by DCB. In addition, most employees are nurses who leave the workforce due to DCB, thereby contributing to staffing shortages [4,70]. Some reports have indicated that the additional costs incurred by DCB exceeded 1 million USD for a 400-bed hospital [71].

Influence Mechanism of DCB

It is necessary that research on the impact of DCB shift from enumeration to elucidating the impact mechanism. The manifestation of DCB is a factor that separates the direct and indirect impact paths. Explicit DCB increases the risk to patient safety, whereas spiteful DCB exacerbates the psychological and social adaptation of victims [32]. In addition, DCB both directly and indirectly threatens patient safety and hospital management. It indirectly impacts patient safety and hospital management by mediating victims’ worsening psychological and social adaptation. The deterioration of victims’ psychological adaptations, such as stress and motivation, decreases their quality of care and their willingness to continue working [72]. Furthermore, discussion on indirect paths regarding communication is limited. Communication problems are caused by poor workplace relationships and account for more than 70% of the adverse events in team medicine [68]. The medical error process has the following three stages: “failure to detect,” “failure to indicate,” and “failure to correct.” However, a communication error in any of these steps results in a medical error without the ability to recover from the failure [73].

Response to DCB

The Johns Hopkins model depicts a component that represents responses to DCB [41]. When a DCB incident occurs, the damage varies depending on how those concerned respond. Similar to coping with stress, individuals respond in four ways, namely, by ignoring, deflecting, confronting, and enlisting [40]. Ignoring, which corresponds to avoidance in stress coping, and deflecting, which corresponds to emotional coping, can prevent the damage from worsening. However, they do not improve the DCB, leading to normalization. Confronting, which corresponds to direct problem-solving, may stop DCB but places a heavy psychological and emotional burden on the victim and may lead to a spiral of conflict. Enlisting, which corresponds to a request for social support, allows an advocate or mediator to stop the DCB. However, there is no guarantee that the advocate or mediator will act appropriately, and they may even become the next victim. It is crucial for the victim(s) to speak up and ask for help in managing DCB. However, an organization’s management often cannot adequately address DCB issues [7]. Influential staff members who possess superior skills and performance and have been with the organization for a long time are more likely to express DCBs. It is not uncommon for employees to hide problems and avoid reporting them, or for the organization’s management to hesitate to address the DCB owing to the perpetrator’s strong influence. Given the organizational countermeasures against DCB discussed in the next section, it is essential to consider that damage occurs to the victim and all stakeholders, including the perpetrator and other team members. In addition, clarifying the different impacts of DCB attributes, such as “spiteful” and “explicit,” is necessary.

**Figure 2:**
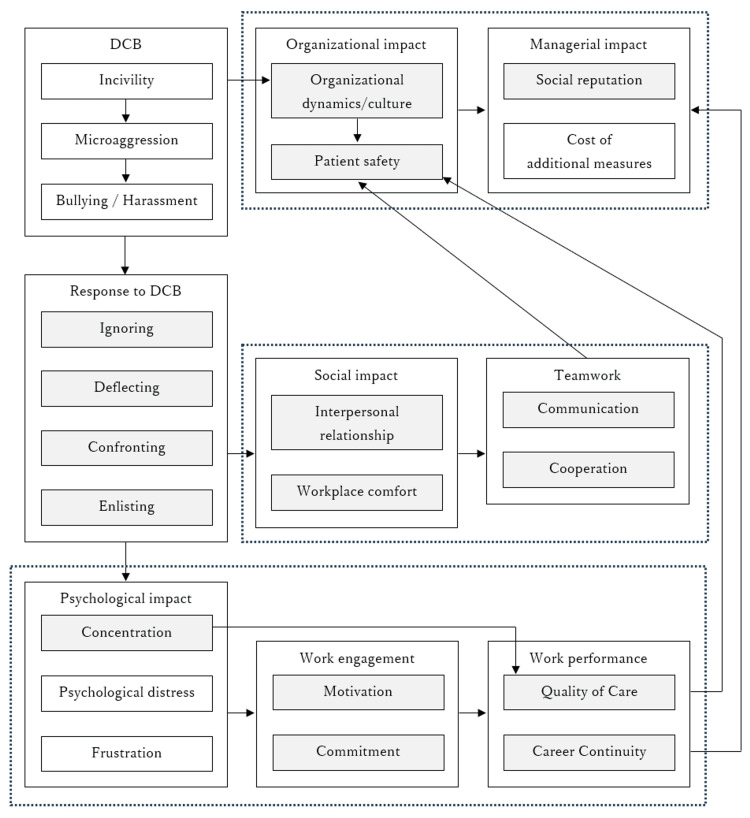
Model organizing findings on the disruptive clinician behavior (DCB) impact mechanism. The dotted boxes represent the organizational, interpersonal, and intrapersonal components from top to bottom. The light grey boxes represent the positive components negatively associated with adverse effects. The figure is original.

Previous studies have examined the impact of DCB. However, some findings suggest multiple non-linear pathways and cascading multistep relationships among these effects. Therefore, to elucidate the impact mechanism, a model should include a direct path in which the responses of the victim and those around them moderate the impact, and the path should terminate in the deterioration of patient safety and organizational management. An indirect path would also be appropriate, in which the victim’s psychological and social state mediates the impact. This can be achieved through a moderated mediation analysis, in which a direct path terminates in the deterioration of patient safety and organizational management, and an indirect path is mediated by the victim’s psychological and social state (Figure 2). A pilot study that tested this model showed that the response partially protected the victim’s psychological and social adaptation by acting as a barrier to the indirect path. However, the prevented harms of DCB pass through the direct path and harm patient safety and organizational management [72]. Therefore, preventing the occurrence of DCB is crucial. Further empirical research in this area is also necessary.

Toward DCB attribution and countermeasures

As shown in Figure 1, the DCB mechanism begins with a trigger that causes the perpetrator’s negative feelings or thoughts [74]. The perpetrator’s argument for their rude behavior may be that the victim is at fault; however, it is still inexcusable for them to express their frustration via DCB. Interpersonal, organizational, and situational problems are likely to have contributed to the non-functioning of the individual’s self-control, meaning that DCB was not discouraged. Therefore, it is not appropriate to “close the curtain” on the problem by overly attributing the cause to the individual perpetrator. It has been noted that the effectiveness of the zero-tolerance principle of countermeasures is limited [75]. In the field of patient safety, which is more advanced than that of DCB [8], the idea of avoiding attributing responsibility for an incident to an individual and letting the entire team share the responsibility is the norm, under the slogan “to err is human.” Although some cases require special handling, such as when the perpetrator has a pathological personality, this idea should be introduced into DCB countermeasures, and the excessive individual sanctions based on “zero-tolerance” should be removed, as everyone makes mistakes. As shown in Figure 2, the harm caused by DCB is not limited to the victim. Suppose that DCB that harms patient safety is considered a type of incident. In this case, DCB should be viewed as a workplace-wide problem, and all stakeholders, including the perpetrator, victim, and those around them, should work to recover from and remedy the harm caused by DCB. A useful concept in this regard is “restorative justice” in conflict resolution. Unlike retributive justice, which focuses on punishing the perpetrator [76], this approach allows stakeholders to understand and discuss the harm through dialogue to restore harmony to the entire community. Concepts and initiatives from other domains, such as restorative justice, should be introduced to advance DCB countermeasures.

The key to DCB contributions and countermeasures is the occurrence mechanism, which states that the risk of DCB occurrence increases when the mind is not at ease (Figure 1). First, countermeasures at the individual level include the improvement of personality and awareness through counseling, psychoeducation, and preventing outbursts of rage through anger control. An essential concept in this context is “nontechnical skills” [77]. Cognitive skills (e.g., situational awareness and decision-making) and task skills (e.g., leadership and task management for task execution) enhance cognitive and task-handling skills and increase mental composure by practicing calm actions even in urgent situations. Relational skills enable individuals to develop and maintain good interpersonal relationships. Personal resource skills, which help maintain good mental and physical health and job motivation, create a sense of well-being. Therefore, training in non-technical skills is expected to have a deterrent effect on DCB. For example, in the patient safety area, there is an organized training program on interpersonal skills called Team STEPPS [78].

Countermeasures at the interpersonal level require all medical staff members to respect and care for each other’s positions and personalities to work comfortably together. This moral commitment is a requirement of professionalism for medical personnel [79]. Medical staff members must curb incivility by recognizing each other’s diversity [80].

Finally, the most critical countermeasure at the organizational level is to improve the working environment. The working environment of medical personnel is harsh, as they work irregular and long shifts, including night shifts, have a heavy responsibility for patients’ lives, and are constantly at risk of lawsuits. Efforts must be made to improve their working conditions by ensuring sufficient staffing and allowing them to work more flexible shifts, thus giving them more time to relax. In addition, social support from supervisors and organizations can effectively reduce the occurrence of DCB [23]. It is also necessary to foster an organizational culture that does not allow DCB to become the norm, for example, by establishing a code of conduct for medical personnel [52].

## Conclusions

We clarified the hierarchical structure of DCB attributes and their mechanisms of occurrence and impact by summarizing the findings of previous studies. We propose the use of a standard definition and terminology for the concept of DCB that considers hierarchical categories. A detailed study of the mediation model in which the reaction to DCB is the adjustment variable should be conducted to elucidate the impact mechanism. Furthermore, examining the specific triggers and impacts of DCB mechanisms at the level of individual behaviors, which are DCB subcategories, is crucial to gaining a deeper understanding of the phenomenon. Changing the concept from “zero-tolerance” to “to err is human” may contribute to proving adequate countermeasures for DCB. As a practical application of these fundamental studies, training programs for DCB contributions and interventions are being developed and verifying their effectiveness based on the accumulated findings.
